# Comparative Chromosomal Analysis of the Z Chromosome in South American Bird Species Shows a High Rate of Intrachromosomal Rearrangements

**DOI:** 10.3390/genes17010112

**Published:** 2026-01-20

**Authors:** Marie Rosellynn C. Enguito, Analía Del Valle Garnero, Ricardo José Gunski, Marcelo Santos de Souza, Rebecca E. O’Connor, Kornsorn Srikulnath, Worapong Singchat, Edivaldo Herculano Correa de Oliveira, Michael N. Romanov, Darren Karl Griffin, Rafael Kretschmer

**Affiliations:** 1 School of Natural Sciences, University of Kent, Canterbury CT2 7NJ, UK; marierosellynndce@gmail.com (M.R.C.E.); rebeckyoc@gmail.com (R.E.O.); m.romanov@kent.ac.uk (M.N.R.); 2 Misamis University, H. T. Feliciano St., Ozamiz City 7200, Philippines; 3 Laboratório de Diversidade Genética Animal, Universidade Federal do Pampa, São Gabriel 97300-000, Brazil; analiagarnero@unipampa.edu.br (A.D.V.G.); ricardogunski@unipampa.edu.br (R.J.G.); 4 Programa de Pós-Graduação em Zoologia, Universidade Federal do Amazonas, Manaus 69077-000, Brazil; marcelodesouzabio@gmail.com; 5 Animal Genomics and Bioresource Research Unit (AGB Research Unit), Faculty of Science, Kasetsart University, Chatuchak, Bangkok 10900, Thailand; kornsorn.s@ku.ac.th (K.S.); worapong.singc@ku.ac.th (W.S.); 6 Instituto de Ciências Exatas e Naturais, Universidade Federal do Pará, Belém 66075-110, Brazil; ehco@ufpa.br; 7 Laboratório de Cultura de Tecidos e Citogenética, SEAMB, Instituto Evandro Chagas, Ananindeua 67030-000, Brazil; 8 L. K. Ernst Federal Research Center for Animal Husbandry, Dubrovitsy, Podolsk Urban Okrug, Moscow Oblast 142132, Russia; 9 Laboratório de Citogenética e Evolução, Departamento de Genética, Instituto de Biociências, Universidade Federal do Rio Grande do Sul, Porto Alegre 91509-900, Brazil; rafael.kretschmer@ufrgs.br

**Keywords:** avian Z chromosome, interspecies fluorescence in situ hybridization (FISH), bacterial artificial chromosome (BAC) probes, South American bird species, intrachromosomal rearrangements, centromere

## Abstract

**Background:** Intrachromosomal rearrangements in birds play a subtle but important role in shaping genomic evolution, phenotypic diversity and speciation. However, the avian sex chromosome system (homogametic ZZ males; heterogametic ZW females) remains relatively understudied, and evolutionary rearrangements of the Z chromosome have not been mapped in most species. To address this, we employed universally hybridizing avian Z chromosome probes to metaphases of 11 avian species from South America. **Methods:** Chromosome preparations were obtained from fibroblast cell cultures of 11 birds representing nine different orders; four bacterial artificial chromosome (BAC) probes were used in our interspecies fluorescence in situ hybridization (FISH) experiments. We identified chromosomal rearrangements in the species investigated, tracing the evolution of the Z chromosome in these species through comparison with reptiles from Southeast Asia (three snake species used as an outgroup), along with two reference species: chicken (Galliformes) and zebra finch (Passeriformes). **Results:** We observed high rates of intrachromosomal rearrangements in the avian Z chromosome, with most species showing different patterns from chicken and zebra finch. *Nannopterum brasilianum* (Suliformes) and *Jacana jacana* (Charadriiformes) showed the same BAC order as chicken, but centromere repositioning was evident. Apart from Piciformes, all other species exhibited a conserved Z chromosome size. The corresponding Z chromosome sequences were homologous to regions of the long arms of Chromosome 2 and W in snakes but not on the Z chromosomes. **Conclusions:** Comparative analysis of the Z chromosome across avian orders provides important insights into the dynamics of avian sex chromosomes and the evolution of sex chromosome systems in general.

## 1. Introduction

Chromosomal organization in birds plays a fundamental role in shaping their genomic evolution, phenotypic diversity and speciation processes [1]. In addition to typically around 36–40 autosomal pairs (~10 macrochromosomes, ~30 microchromosomes), a ZW sex chromosome system predominates [2].

Recent reviews by Griffin et al. [3,4] showed that, although avian genome organization is largely conserved and dates back to the age of the dinosaurs, some orders exhibit highly derived karyotypes. For example, Falconiformes (falcons, hobbies and relatives) have a reduced diploid number of approximately 50 chromosomes [5], whereas Piciformes (woodpeckers, toucans and relatives) display strikingly high diploid numbers exceeding 100 chromosomes [3,4,6,7]. Recent large-scale studies demonstrate that phylogenomic signals differ between micro- and macrochromosomes and that assigning loci to specific chromosomes improves the resolution of complex evolutionary histories [8,9]. For instance, there are contrasting roles for microchromosomes and macrochromosomes [10], the former being gene-rich, GC-rich, tending to higher recombination rates, and evolving faster than macrochromosomes [11]. Patterns of lineage-specific chromosomal rearrangement thus not only illustrate exceptions to long-term karyotype stability but also set the stage for further structural innovations that have profound effects on avian evolution [12]. This emphasizes the fact that a detailed understanding of chromosomal organization is essential for interpreting genome evolution and for reconstructing avian evolutionary trees accurately [13].

Beyond their value for phylogenetic reconstruction, patterns of chromosomal organization actively shape how genetic variation is generated and maintained across avian genomes. Recent cytogenomic and comparative genomic studies demonstrate that chromosomal rearrangement, variation in recombination rate, and sex chromosome dynamics jointly drive lineage-specific evolutionary trajectories in birds [14,15,16]. For instance, inversions and other intrachromosomal rearrangements can suppress recombination in affected regions, allowing co-adapted allele combinations to persist [17]. Indeed, inversion polymorphisms often coincide with loci implicated in local adaptation and reproductive isolation. This indicates that chromosomal structure can actively promote divergence and, in some cases, speciation by preserving suites of adaptive alleles from recombination-mediated breakup [18].

Moreover, the propensity for structural chromosomal changes is modulated by genomic features that influence chromosome stability. Transposable element activity, repeat density and the presence or absence of DNA-repair genes can bias lineages toward higher or lower rates of rearrangements. For instance, parrot lineages with altered repair or gene-loss signatures show elevated fusion and fission activity, helping explain why some bird clades remain highly karyotypically stable, while others are remarkably labile [18]. Collectively, the partitioning of genomes into micro- and macrochromosomes, lineage-specific rearrangement patterns and, relevant to this study, the behavior of the Z and W sex chromosomes act as a structural scaffold for the mapping of aspects of phenotypic trait evolution. These include determining where and how variation arises, how it is shuffled by recombination, how it responds to natural selection, adaptation rates and the emergence of sex-linked phenotypes.

As reviewed by Griffin et al. [3,4], the avian ZW sex-determination system was probably in place before the divergence of Palaeognathae and Neognathae, but the size differentiation between Z and W chromosomes emerged after this divergence. In the ZW sex chromosomes of most Neognathae, the W chromosome is dynamic, both in size and morphology, because of the accumulation of repetitive sequences [19,20,21]. The Z chromosome, however, unlike the W chromosome, which has undergone extensive degeneration, shows relatively conserved gene content, with structural rearrangements such as inversions comprising most of its variation [2,3,4]. Typically, the Z chromosome is usually around the fifth-largest chromosome and conserved in size. However, there are exceptions; in Piciformes, it is unusually large, probably because of the accumulation of repetitive DNA sequences [6,22]. Indeed, it exhibits notable morphological variability even among closely related species. For instance, in Columbidae (pigeons, doves, etc.), *Columbina picui* (Picui ground dove) possesses a telocentric Z chromosome, whereas *Leptotila verrauxi* (white-tipped dove) has a metacentric Z. This highlights the role of intrachromosomal changes in its evolution [23]. Similarly, fluorescence in situ hybridization (FISH) mapping using a bacterial artificial chromosome (BAC) clone (for the *ALDOB* gene) across four Galloanseres species (*Gallus gallus*, *Coturnix coturnix*, *Chrysolophus pictus*, and *Cairina moschata*—chicken, common quail, golden pheasant, and muscovy duck) revealed species-specific signal patterns [24], with BAC-FISH analysis also being instrumental for refining centromere positions [25].

Recent reviews suggest that avian sex chromosomes undergo considerable inter- and intrachromosomal change and are thus not as stable as previously thought [1,2,3,4,10]. For instance, a multiple-sex chromosome system (♂Z1Z1Z2Z2/♀Z1Z2W) has been described by Gunski et al. [26] in the Adélie penguin (*Pygoscelis adeliae*, Sphenisciformes) and in *Sula* species [27]. Independent autosome–sex chromosome fusions have also been reported in Sylvioidea species [28,29,30,31,32,33,34] and neo-sex chromosomes derived from such fusion events have been identified in parrots, cuckoos and Pacific Island birds [18,35,36,37]. Neo-sex chromosomes can diverge rapidly in gene content and expression; they can influence sexual dimorphism and affect patterns of selection because of sex-specific inheritance and suppressed recombination [29,32].

Given that evolutionary rates and rearrangement frequencies vary significantly by chromosome [38], genomic analyses that ignore the chromosomal context risk introducing bias. The Z chromosome is particularly critical in this regard. That is, while its gene content is conserved, its structural rearrangements can act as powerful drivers of reproductive isolation and phenotypic evolution. Therefore, characterizing these Z-specific structural patterns is essential for accurate phylogenomic interpretations [12]. This study addresses this need by investigating the role of Z chromosome rearrangements in the diversification of the class Aves, providing new insights into the evolutionary consequences of sex chromosome variability in South American birds.

Indeed, despite several studies, the extent and diversity of chromosomal rearrangements in avian Z chromosomes remain poorly explored. This is partly due to limited molecular cytogenetic tools compared to the autosomes [4,39]. A critical gap in our understanding of avian karyotype evolution is therefore apparent. South American birds particularly represent distinct evolutionary radiations, with diverse ecological pressures. Their Z chromosome organization nonetheless remains poorly characterized [22,40]. This study thus applied four universally hybridizing FISH probes derived from chicken and zebra finch genomes to the Z chromosomes of 11 South American bird species representing nine distinct orders. Comparative analysis with data from squamate reptiles (snakes) plus chicken and zebra finch provides further insight into the evolutionary origins and diversification of the avian Z chromosome, tracing its structural evolution across different lineages.

## 2. Materials and Methods

### 2.1. Sample Collection

Metaphase chromosome preparations were obtained from 11 bird species (one per species) from nine different orders (Table 1), along with, for comparison, two reference avian species (chicken and zebra finch) as well as three outgroup reptiles (snakes) (Table 1). Specimens were sourced ethically from research collaborations, ensuring minimal impact on wild populations. Tissue samples (blood or fibroblast cultures) were collected following approved institutional animal care and use protocols.

### 2.2. Chromosome Preparation

Primary cell cultures were established from tissue samples using standard protocols. Mitotic cells were arrested at metaphase by colcemid (0.05 μg/mL) exposure for 1 h, followed by hypotonic treatment in 0.075 M KCl for 20 min to cause swelling in the cytoplasm and the nucleus, making the nuclear membrane more fragile for the release of chromosomes, generating clear metaphase spreads. Cells were fixed in a 3:1 methanol/acetic acid solution (Carnoy’s fixative) to preserve cellular and chromosomal structure, and metaphase spreads were prepared on clean glass slides following previously published protocols [19]. Chromosomes were air-dried and stored at −20 °C until use. The chromosome preparations used here were the same as in previous studies shown in the tables presented above.

### 2.3. Fluorescence In Situ Hybridization (FISH)

For interspecies chromosome mapping, we selected four BAC probes derived from the Z chromosome of chicken, *Gallus gallus* (CH261-129A16 and CH261-133M4), and zebra finch, *Taeniopygia guttata* (TGMCBA-200J22 and TGMCBA-27019), which showed higher hybridization efficiency in cross-species FISH experiments [39]. These probes were used to investigate intrachromosomal rearrangements. Chromosomes were counterstained with DAPI (blue), and the BAC probes were labeled with Texas Red (red) or FITC (green). At least 10 metaphases per experiment were analyzed to confirm the FISH results. The BAC clone isolation, amplification, labeling and FISH followed O’Connor et al. [49]. This approach has been applied routinely in our respective laboratories for many years, and we used it without adaptation.

### 2.4. Imaging and Data Analysis

Chromosomes were counterstained before analysis with 4,6-diamidino-2-phenylindole (DAPI). FISH signals and DAPI-stained chromosomes were photographed using an Olympus BX61 epifluorescence microscope equipped with a cooled CCD camera (Olympus, Tokyo, Japan), and the images were captured at 100× magnification using SmartCapture 3 software (Digital Scientific UK, Cambridge, UK) [49]. Comparative analysis of signal patterns among South American birds, as well as with published data from squamate reptiles, was used to infer evolutionary rearrangements and the structural dynamics of the Z chromosome.

### 2.5. Comparative Analysis and Evolutionary Inference

Intrachromosomal rearrangements were categorized according to type and frequency across species, using BAC probe positions on the chicken Z chromosome as a reference. Homology comparisons with previously published FISH data on reptiles provided context for the origin and evolution of the avian Z chromosome. Observed rearrangements were interpreted in the framework of lineage-specific chromosomal evolution, sex chromosome diversification, and potential adaptive significance.

## 3. Results

### 3.1. Comparative FISH Mapping Across South American Birds

**Figure 1:**
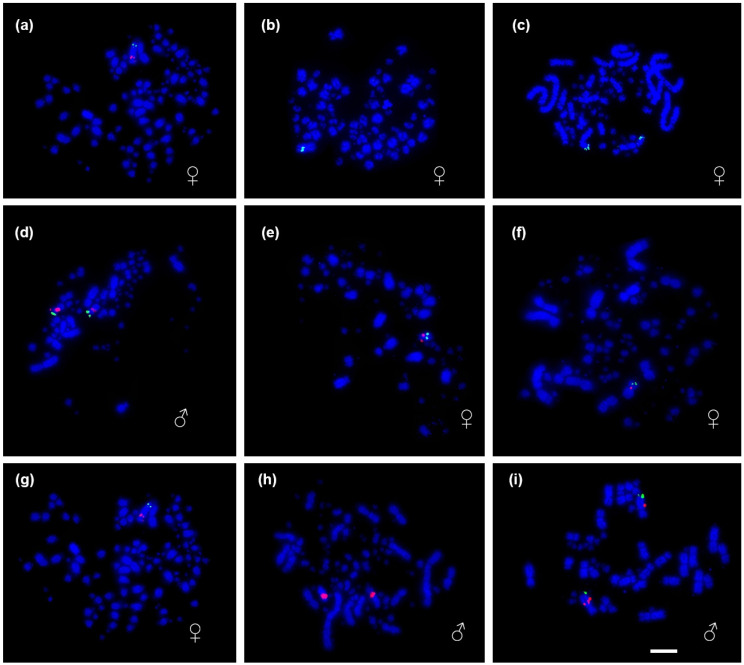
FISH mapping of Z chromosome BAC probes onto metaphase of selected South American bird species. The panels show hybridizations for: (**a**) female (*V. spilogaster*) with zebra finch BAC probe TGMCBA-200J22 (green); (**b**) female (*V. spilogaster*) with chicken BAC probe CH261-129A16 (green) and zebra finch BAC probe TGMCBA-27019 (red); (**c**) male (*N. brasilianum*) with zebra finch BAC probe TGMCBA-200J22 (green); (**d**) male (*N. brasilianum*) with chicken BAC probes CH261-129A16 (green) and CH261-133M4 (red); (**e**) female (*H. torquata*) with zebra finch BAC probe TGMCBA-27019 (red) and chicken BAC probe CH261-133M4 (green); (**f**) male (*G. melanops*) with chicken BAC probe CH261-129A16 (green) and zebra finch BAC probe TGMCBA-27019 (red); (**g**) female (*P. inscriptus*) with chicken BAC probe CH261-129A16 (green) and zebra finch BAC probe TGMCBA-27019 (red); (**h**) male (*J. jacana*) with chicken BAC probe CH261-133M4 (red); and (**i**) male (*M. monachus*) with chicken BAC probe CH261-129A16 (green) and zebra finch BAC probe TGMCBA-27019 (red). In all cases chromosomes are counterstained with 4′6′ diamidinophenylindole (DAPI)—blue colour. Scale bar = 10 µm.

**Figure 2:**
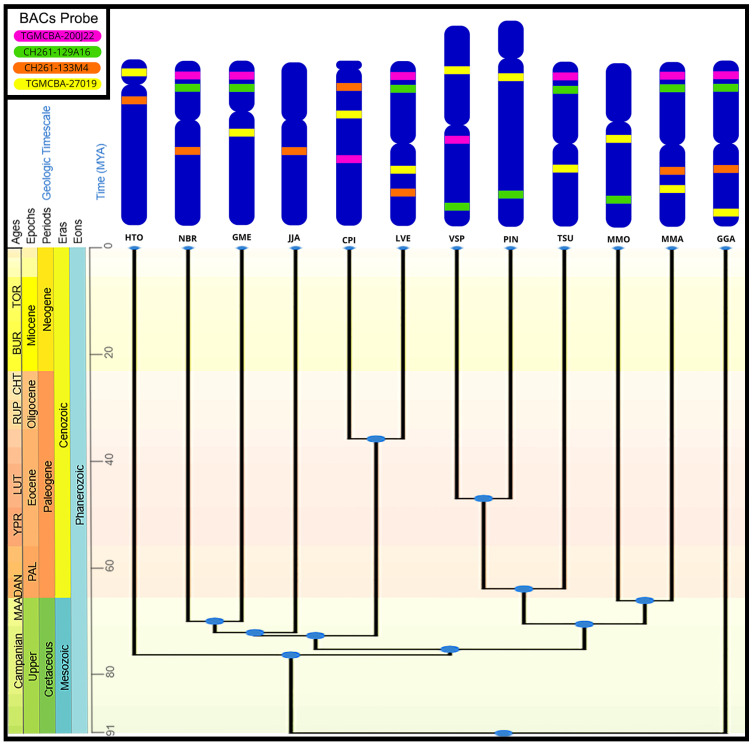
Summary of the chromosome positions of each of the BACs established by manual measurement of fractional length from the p-terminus (FLpter). Ideograms represent the Z chromosomes, arranged according to taxonomic proximity, with colored bands indicating the mapped positions of four BAC probes. The probes utilized were derived from chicken (*G. gallus*; CH261-129A16 and CH261-133M4) and zebra finch (*T. guttata*; TGMCBA-200J22 and TGMCBA-27019) genomes. The adapted phylogenetic tree was sourced from TimeTree databases (http://www.timetree.org, accessed on 7 December 2025). Species names are represented by individual code indicated below each respective ideogram: LVE (*L. verreauxi*), CPI (*C. picui*), HTO (*H. torquata*), TSU (*T. surrucura*), NBR (*N. brasilianum*), GME (*G. melanops*), JJA (*J. jacana*), VSP (*V. spilogaster*), PIN (*P. inscriptus*), MMO (*M. monachus*), MMA (*M*. *maculatus*).

There were 33 probes successfully hybridized onto the metaphases of 11 South American bird species. When probes did not show signals, this was down to insufficient number of metaphases or unknown factors (we find this to be commonplace in work of this kind). Our results nonetheless provide evidence of a high rate of intrachromosomal rearrangements in the avian Z chromosomes of South American birds, with most species showing different patterns from chicken and zebra finch. The species exhibited all possible chromosomal morphologies for the Z chromosome (metacentric, submetacentric, acrocentric, and telocentric), as shown in Figure 1 and Figure 2.

### 3.2. Mapping Intrachromosomal Changes with Avian BAC Probes

Table 2 shows comparative mapping of chicken Z chromosome BAC probes onto the chromosomes of *N. kaouthia* (monocled cobra). The chromosomes in *N. kaouthia* where the BAC probe hybridized were the long arm of W chromosome (Wq) and the long arm of chromosome 2. Parts of the chicken Z chromosome correspond to different chromosomes in *N. kaouthia*. Some Z-linked BACs mapped to W chromosome (long arm), suggesting sex chromosome homology. Other Z-linked BACs mapped to autosome 2 (long arm), indicating chromosomal rearrangements (like translocations) between species.

## 4. Discussion

### 4.1. Evolutionary Insights into the Avian Z Chromosome

The overall evolutionary dynamics of Z chromosome rearrangement in the species studied are summarized in Figure 2. This gives a global overview of how the Z chromosome has changed intrachromosomally, despite being conserved interchromosomally. Of course, there were limitations inherent in our approach. They included the fact that not all probes worked on all species, the limits of resolution of all molecular cytogenetic studies (even on very extended chromosomes), the labor-intensive nature of the work, and the fact that it all relies on the vagaries of cell culture to generate analyzable metaphases.

Of the 11 species tested, only two, *N. brasilianum* and *J. jacana*, showed the same BAC order as chicken (Figure 2). These, however, were submetacentric chromosomes, different from those of the chicken (which has a metacentric morphology), evidencing the occurrence of a centromere repositioning. The other species showed different chromosome positions, at least for one Z BAC probe. The exception was in the Piciformes, which have the Z as the largest chromosome in the karyotype, the other species having a conserved Z chromosome size (about the fifth largest). The morphology of this chromosome is variable in birds, even in closely related species. For instance, the Z chromosome in *C. picui* is telocentric, while it is metacentric *in L. verrauxi* [23]. Moreover, the chicken Z chromosome is partially conserved in cobra species but reorganized across chromosomes. Two BACs (CH261-129A16 and TGMCBA-200J22) mapped to Wq. This suggests that regions of the chicken Z are homologous to the cobra W chromosome, hinting at shared ancestral sex chromosome segments. The other two BACs mapping to 2q show intrachromosomal or interchromosomal rearrangements, supporting the idea that bird and snake genomes have undergone major rearrangements since their divergence. This result helps reconstruct evolutionary changes in sex chromosomes and autosomes between birds and snakes.

Most studies now suggest that chicken has a genome organization most closely related to the ancestor [50,51,52]. If we consider this as the avian outgroup, *N. brasilianum*, *M. maculatus* and *J. jacana*, having the same BAC order as chicken, provide evidence that there were no detectable intrachromosomal rearrangements but a repositioning of the centromere (Figure 2). For each of the other species, a combination of intrachromosomal rearrangements and centromere repositioning led to the configurations seen (Figure 2).

### 4.2. Reptilian Origins

Our results support a model in which the chicken Z chromosome shares ancestral chromosomal segments with both the cobra W chromosome and autosome 2. This reflects both deep homology and extensive post-divergence genomic reorganization. Such findings contribute to reconstructing the evolutionary history of sex chromosomes in amniotes, demonstrating that the avian Z chromosome is not evolutionarily isolated but retains signatures of ancient chromosomal structures shared with reptiles [3]. This aligns with recent comparative studies showing that amniote sex chromosomes have evolved through repeated turnovers, partial homologies and independent recruitment of autosomal segments into sex chromosome systems [53]. Integrating cytogenetic mapping with comparative genomics [54,55] therefore provides a powerful framework to trace the origin and transformation of sex chromosomes across vertebrate evolution [56,57,58]. Cross-referencing with published whole-genome alignments shows that the BAC signals on squamate W and chromosome 2 match conserved syntenic regions previously identified as homologous to parts of the avian Z chromosome. O’Connor et al. [59] suggested that, at least in some turtles (which are phylogenetically closer to birds than snakes), the Z chromosome and chromosome 6 (designated as an autosome, as there is no evidence of a ZZ/ZW system) are homologous. Genome alignments of other turtles, however, indicate the absence of a strict 1:1 relationship. These data imply that the avian Z chromosome, as currently defined, originated around the time of the bird–turtle divergence and, with few exceptions, has remained largely conserved since then [60]. The avian Z sequence mapped to the squamate W rather than the Z, likely due to lineage-specific rearrangements. Despite overall degeneration, the W chromosome can retain ancestral fragments, whereas the corresponding region on the squamate Z was probably lost or reorganized after divergence. The forces of evolution therefore seem to have favored the absence of interchromosomal rearrangement but not intrachromosomal rearrangement. Indeed, O’Connor et al. [59] suggested that the Z chromosome (and most others) was present intact in non-avian dinosaurs.

### 4.3. Intrachromosomal Rearrangement Predominates

Our findings further support the view that the avian Z chromosome evolves through a higher rate of intrachromosomal rearrangements than autosomes. This is reflected by differences in BAC order and/or centromere position in most species analyzed, relative to chicken and zebra finch. Moreover, the Z chromosomes show every major morphological class across the sample (metacentric, submetacentric, acrocentric, telocentric). This demonstrates that, while avian genomes are relatively conserved at the autosomal karyotypic level, Z chromosomes are prone to lineage-specific internal reorganization [35]. The conserved BAC order, yet different chromosome morphology, in *N. brasilianum*, *M. maculatus* and *J. jacana* invokes a mechanism of centromere repositioning. This is increasingly recognized as an important mechanism in avian chromosome evolution, allowing changes in chromosomal morphology independent of large-scale rearrangements [39]. The remaining species, most likely, arose because of intrachromosomal rearrangements such as inversions, transpositions or localized rearrangements. Such rearrangements can accumulate over evolutionary time and have been frequently documented in the avian Z chromosome across multiple orders [61,62,63]. Inversions on Z chromosomes have been previously documented and shown to affect recombination and phenotypes in passerines and other groups. Centromere shifts can convert telocentric chromosomes into (sub)metacentric forms without large-scale exchange of genetic material [64]. These processes are also often associated with the accumulation or redistribution of repetitive elements and retrotransposons on sex chromosomes, which create substrates for non-allelic homologous recombination and breakpoint formation [64]. This compares to alignments of chromosome-level genome assemblies and BAC mapping in multiple Asian species that revealed inversions affecting macrochromosomes, including the Z [65]. In contrast, Neotropical chromosome-scale assemblies reveal lineage-specific rearrangements and centromere shifts in New World taxa, even though they belong to different orders. Together, these data indicate that the processes generating Z-morphological diversity are widespread geographically and not restricted to a single avian region or clade [65]. The unusually large Z chromosome in the Piciformes reflects lineage-specific expansion in chromosome size because of accumulation of repetitive DNA, which has already been reported in other groups as well [18]. Such changes may have functional implications for gene content, recombination landscapes, and even dosage compensation mechanisms [66].

### 4.4. Functionally and Evolutionarily, Intrachromosomal Rearrangements on the Z Chromosome Have Three Important Features

First, inversions and centromere shifts can modify recombination landscapes along the Z. This can alter linkage relationships among Z-linked genes, thus facilitating the joint inheritance of adaptive alleles or the buildup of sex-linked differentiation. This matches recent cytogenomic and population-genomic work showing that the avian Z undergoes faster structural change than many autosomes and often accumulates inversions and centromere shifts that change recombination landscapes and local gene order. Such intrachromosomal events can explain why even closely related species differ in Z chromosome BAC order while conserving overall gene content. These rearrangements have been documented in multiple recent bird studies and are increasingly recognized as a common mechanism driving lineage-specific Z differentiation [67].

Second, changes in gene order/position can cause position effects or alter regulatory contexts for dosage-sensitive Z genes [68]. These may have downstream impacts on sexually dimorphic traits. Hybridization of avian Z BACs to reptile metaphases provides direct cytogenetic evidence that parts of the modern avian Z derive from ancestral autosomal linkage groups that are still present, in whole or in part, in reptile genomes. The localization of BAC clones on chromosome 2 in these squamate species aligns with broader comparative mapping studies, indicating that many amniote sex chromosomes stem from a restricted set of ancestral autosomes. This suggests that different lineages have repeatedly recruited and reused overlapping genomic blocks into sex chromosomes, producing partial homologies. The avian Z is thus a mosaic assembled from ancestral autosomal segments that were differentially retained, inverted, or translocated in birds but, importantly, were retained interchromosomally because, in part, of their role in sexual dimorphism [4]. Our data therefore fit a growing consensus that sex chromosomes evolve through repeated reuse and rearrangement of a limited set of ancestral genomic building blocks [66].

Third, because the Z is primarily transmitted through males, structural changes that reduce recombination or create incompatibilities may contribute to reproductive isolation and lineage divergence [63]. Recent syntheses emphasize that sex chromosome recruitment and turnover across amniotes is non-random. That is, certain linkage groups are predisposed to sex linkage, and centromere repositioning and repeat accumulation commonly accompany these transitions. Intrachromosomal rearrangements on the Z alter recombination and linkage relationships, which can facilitate the fixation of sexually beneficial alleles, influence dosage compensation trajectories and contribute to reproductive isolation.

Detecting Z-homologous segments in reptiles provides testable hypotheses regarding ancestral gene content and which Z-linked genes might have been important early in avian sex chromosome evolution. Future work that combines BAC mapping, chromosome-scale assemblies from many regions, and gene-level analyses in the pertinent reptile taxa will enable timing of specific rearrangements and assessment of their phenotypic and evolutionary impact.

## 5. Conclusions

This study demonstrates that intrachromosomal rearrangements of the Z chromosome are a significant factor in avian genome evolution. By linking chromosomal structure to phylogenetic divergence, our findings provide a framework for future research aimed at integrating cytogenomics with molecular and morphological data. This will help reconstruct the evolutionary tree of birds with higher resolution and greater accuracy. The comparative chromosomal analysis of the Z chromosome in South American bird species reveals a notably high rate of intrachromosomal rearrangements, indicating that the Z chromosome is more structurally dynamic than previously assumed. That is, while avian karyotypes are generally conserved, the observed rearrangements suggest that sex chromosomes, particularly the Z chromosome, are hotspots for genomic innovation and divergence. The observation that the Z chromosome exhibits all possible morphologies—metacentric, submetacentric, acrocentric and telocentric—across the studied species reinforces notions of its high structural plasticity. Comparative mapping of chicken Z chromosome BAC probes onto snake chromosomes demonstrates that the avian Z is partially conserved but extensively reorganized in the reptile–bird divergence. This study thus provides valuable cytogenetic evidence for reconstructing the evolutionary origins and transformations of avian sex chromosomes in the context of broader amniote genome evolution. These structural changes may contribute to species-specific adaptations, influence the evolution of sex-linked traits, potentially affect reproductive isolation and ultimately contribute to the processes of speciation.

## Figures and Tables

**Table 1:** Species used in this study, Latin name, common name, species code, shape of chromosome, diploid chromosome number and most appropriate reference with an accurate chromosome number. Arranged according to evolutionary tree, e.g., Passeriformes are most distant from Squamata.

Order	Scientific Name	3-Letter Species Code	Common Name	Z Morphology	2n	Reference
**New South American avian species**						
Passeriformes	*Myiodynastes maculatus*	MMA	Streaked Flycatcher	Metacentric	80	[41]
Psittaciformes	*Myiopsitta monachus*	MMO	Monk Parakeet	Submetacentric	48	[42]
Piciformes	*Veniliornis spilogaster*	VSP	White-spotted Woodpecker	Metacentric	88	[43]
Piciformes	*Pteroglossus inscriptus*	PIN	Lettered Araçari	Acrocentric	112	[6]
Trogoniformes	*Trogon surrucura*	TSU	Surucua Trogon	Metacentric	82	[7,44]
Suliformes	*Nannopterum brasilianum*	NBR	Neotropical Cormorant	Submetacentric	74	[7]
Charadriiformes	*Jacana jacana*	JJA	Wattled Jacana	Submetacentric	82	[35,45]
Gruiformes	*Gallinula melanops*	GME	Spot-flanked Gallinule	Submetacentric	80	[20]
Caprimulgiformes	*Hydropsalis torquata*	HTO	Scissor-tailed nightjar	Acrocentric	74	[7]
Columbiformes	*Leptotila verreauxi*	LVE	White-tipped Dove	Metacentric	78	[23]
Columbiformes	*Columbina picui*	CPI	Picui Ground Dove	Telocentric	76	[23]
**Reference species**						
Passeriformes	*Taeniopygia guttata*	TGU	Timor Zebra Finch	Metacentric	80	-
Galliformes	*Gallus gallus*	GGA	Red junglefowl	Metacentric	78	-
Squamata	*Lacerta agilis*	LAG	Sand lizard	Acrocentric	38	[46]
Squamata	*Daboia russeli*	DRU	Western Russel’s Viper	Metacentric	36	[47]
Squamata	*Notechis scutatus*	NSC	Mainland Tiger Snake	Metacentric	36	[47]
Squamata	*Naja kaouthia*	NKA	Siamese cobra	Metacentric	38	[48]

**Table 2:** Comparative mapping of chicken Z chromosome BAC probes onto the chromosomes of *Naja kaouthia*.

Chromosome Number	Chicken and ZF Chromosome	BAC Name	Chromosomal Location in *Daboia russeli*	Chromosomal Location in *Notechis scutatus*	Chromosomal Location in *Naja kaouthia*
40	Z	CH261-129A16		-	Wq
41	Z	TGMCBA-200J22		W	Wq
42	Z	TGMCBA-27019	W	W	2q
43	Z	CH261-133M4		2p, W	2q

## Data Availability

All raw data is available by contacting the authors.

## Abbreviations

The following abbreviations are used in this manuscript:

FISH	Fluorescence in situ hybridization
BAC	Bacterial artificial chromosome
KCl	Potassium chloride
DAPI	4,6-diamidino-2-phenylindole
